# Stemming the tide: promoting mental health and preventing mental disorders in low- and middle-income countries

**DOI:** 10.1017/gmh.2015.9

**Published:** 2015-07-08

**Authors:** W. A. Tol

**Affiliations:** 1 Department of Mental Health, Johns Hopkins Bloomberg School of Public Health, 624 N Broadway, Baltimore, MD, USA; 2 Program Director, the Peter C. Alderman Foundation, Bedford, NY, USA

**Keywords:** mental health, low- and middle-income countries, mental health promotion, prevention, global mental health, public mental health

## Abstract

The first World Health Organization's global action plan for mental health recognizes the importance of mental health promotion and prevention of mental disorders, through the inclusion of one of four objectives focused on this crucial area of research and practice. This paper aims to provide an ‘aerial view’ of the field of mental health promotion and prevention of mental disorders with a focus on low- and middle-income countries. Starting with reasons why promotion and prevention need to take center stage in global mental health efforts, the paper provides a framework and four general principles to guide such efforts: a socio-ecological perspective (place); an inter-sectoral and interdisciplinary approach (collaboration), a developmental perspective (timing), and a participatory and empowerment approach (strengths), or PaCTS. Evidence-based examples of mental health promotion, universal, selective, and indicated prevention are described.

It is time for promotion and prevention efforts to take center stage in the field of global mental health. The inclusion of an objective focused specifically on strategies for promotion and prevention in the first WHO global action plan for mental health (World Health Organization, 2013), is a timely occasion to reflect on what is known about effective interventions, and what the key questions are. This paper aims to provide an ‘aerial view’ of the field of mental health promotion and prevention of mental disorders with a focus on low- and middle-income countries (LMICs). The range of options for action in this field is indicated by highlighting successful interventions that had diverse aims and were implemented across a range of contexts.

Given the breadth of this topic and limited space, the scope of this paper is restricted in several ways. First, when discussing prevention efforts, the focus will be on what are often referred to as the ‘common’ or ‘non-psychotic’ mental disorders (including depression, anxiety, medically unexplained symptoms, and posttraumatic stress symptoms) – disorders such as psychosis and bipolar disorder are not discussed, nor is suicide. This is not to indicate that fruitful prevention efforts for these conditions do not exist (e.g. see van der Feltz-Cornelis *et al*. 2011; Stafford *et al*. 2013). Also, the focus is on promotion and prevention aimed at the social determinants of health, rather than in the domain of biology, e.g. through pharmacological methods, diet supplements or considering gene-environment interactions. Again, this is not to neglect the wealth of possibilities that are starting to become apparent in this area (Southwick & Charney, 2012). Third, where possible the discussion focuses on opportunities for action that have been shown to be associated with benefits in rigorous intervention outcome research.

## Definitions

Mental health is commonly defined as ‘a state of wellbeing in which an individual realizes his or her own abilities, can cope with the normal stresses of life, can work productively and is able to make a contribution to his or her community’ (World Health Organization, 2004). This definition invokes a broader notion of optimal psychological and social functioning, and not just the absence of psychological symptoms and mental disorders. The term ‘mental health promotion’ is tied to this use of the concept of mental health, as it is generally aimed at strengthening positive aspects of mental health, for example promoting self-esteem in adolescents or strengthening positive coping skills in school-aged children (Barry *et al*. 2013; Fazel *et al*. 2014). In contrast, prevention focuses on mental disorders, in that it is aimed at ensuring that mental disorders do not develop. Prevention in turn differs from treatment, as treatment is focused on assisting people with identified mental disorders.

The Institute of Medicine has proposed a useful framework for promotion and prevention in the field of mental health (National Research Council & Institute of Medicine, 2009). This framework builds on the classification developed by Gordon (1983) and divides prevention into universal, selective, and indicated prevention. Universal prevention refers to interventions with the whole population, regardless of risk and protective factors, for example a mass media campaign aimed at providing information on alcohol abuse. Selective prevention entails interventions with populations exposed to a specific risk factor, for example relaxation exercises in schools or workplaces particularly affected by an earthquake. Indicated prevention targets individuals who may have signs of a mental disorder, but who do not meet a diagnosis, or who have a biological marker for a specific disorder, for example parent-training interventions for children with early aggressive behaviors (National Research Council & Institute of Medicine, 2009).

## Why focus on prevention and promotion in global mental health?

There are a number of reasons why promotion and prevention efforts should be central to mental health policies and programs. First, there are limitations to what a strategy focused on treatment alone can offer. Given the current burden of mental disorders, it is unlikely that enough therapists can be trained to completely reduce the gap between people in need of services and the services that are available, also if applying a task sharing model. For example, Andrews *et al*. (2000) estimated that with maximum coverage of current services in Australia, only 36% of the burden of depression can be treated.

Second, the global mental health movement has been criticized for placing too much emphasis on the treatment of mental disorders, when there is evidence that social conditions play an important role in the genesis of mental disorders (Kirmayer & Pedersen, 2014). Although social conditions, for example poverty, have been of interest to global mental health researchers and practitioners (World Health Organization, 2001, 2004; Patel & Kleinman, 2003; Herrman *et al*. 2005; Patel *et al*. 2007; Lund *et al*. 2011; World Health Organization & Calouste Gulbenkian Foundation, 2014) and prevention has been a key item in global mental health research agendas (Tomlinson *et al*. 2009; Collins *et al*. 2011), scaling-up treatments appears to remain the key focus (Lancet Mental Health Group, 2007).

It is important to note that there are important synergies between treatment and prevention (Patel, 2014). For example, cognitive behavioral treatments have been shown to reduce physical and psychological intimate partner violence (Tirado-Munoz *et al*. 2014), a key risk factor for various mental disorders. In the same vein, treatment of mental disorders in caregivers may contribute to improved indicators of child development (Petersen *et al*. 2011). Nevertheless, it seems irresponsible to treat mental disorders in contexts of chronic poverty or violence without also throwing ones full weight behind proactive efforts to reduce these structural forces that bring people to health care clinics in the first place. Clearly, this makes pragmatic sense from a public health perspective. In addition, addressing the social determinants of mental health is essential from a rights-based perspective. Addressing the social determinants of mental health is critical to address health inequities (i.e. the fact that health risks and the burden of ill health are unfairly distributed across populations) – a hallmark interest of global health in general (Koplan *et al*. 2009). Bringing prevention and promotion research and intervention to the heart of the global mental health agenda would contribute to address calls for a more inclusive global mental health field that pays attention to macro-level socio-cultural processes rather than approaching mental health purely as a technical biomedical problem to be solved through globally uniform solutions (Kirmayer & Pedersen, 2014).

A third reason for why promotion and prevention should be of central interest to global mental health is that cost-effective interventions are available. There is an existing body of research supporting the effectiveness of interventions aiming to prevent mental disorders and promote mental health. A recent review of mental health promotion and universal prevention found 46 studies employing randomized controlled trial or quasi-experimental designs in LMICs (Barry *et al*. 2011). For children and adolescents specifically, 22 studies were identified: 14 focused on school-based interventions, with overall strong quality of evidence; and eight studies on community-based interventions, with moderate to strong quality of evidence (Barry *et al*. 2013). Economic analyses are starting to emerge (Knapp *et al*. 2011; Roberts & Grimes, 2011). Evidence of cost-effectiveness is promising, but limited to a smaller number of interventions with restricted scope for generalizability across socio-cultural settings. Evidence of cost-effectiveness is most promising for early childhood development programs (Zechmeister *et al*. 2008). Overall, more evaluation research is necessary to cover interventions across the breadth of this field, but enough evidence exists to enable action now.

## Principles for action

Four key principles may be distilled from the current literature on prevention and promotion in mental health in LMICs (see Box 1) (for helpful overviews, see Petersen *et al*. 2010, 2014; Beardslee *et al*. 2011; World Health Organization, 2012; World Health Organization & Calouste Gulbenkian Foundation, 2014). First, since mental health is shaped by the context in which people live, efforts need to target known modifiable risk, protective, and promotive factors for mental health in the social environment. These are existent at the individual level (e.g. coping styles and self-esteem), as well as at family- (e.g. parental mental health and parenting styles), peer- (e.g. peer pressure and social support), school- or work place- (e.g. opportunities for skill development), and community-levels (e.g. social capital and communal violence). A socio-ecological framework has been helpful in disentangling the reciprocal influences between the individual and their environment, providing insight into which social variables may be targeted to promote mental health and prevent mental disorders (Bronfenbrenner, 1994; Lynch & Cicchetti, 1998).

Box 1. PaCTS – core principles for the promotion of mental health and prevention of mental disorders in *LMICs*

(1) *Place*. A socio-ecological perspective.
(2) *Collaboration*. An inter-sectoral and interdisciplinary perspective.
(3) *Timing*. A developmental perspective.
(4) *Strengths*. Community-based and empowering.

Second, prevention and promotion interventions need to take into account a developmental perspective. Since almost half of lifetime mental disorders start before age 14 (and up to 75% before age 24), childhood and adolescence are important periods to start prevention and promotion programs (Beardslee *et al*. 2011). Given that development proceeds sequentially, with later skills dependent on successful acquisition of earlier skills, the early childhood period has been advocated as a particularly cost-effective period for intervention (Heckman, 2006). At the same time, a developmental perspective entails viewing different developmental stages – throughout the life span – as periods with unique confluences of risk, protective, and promotive factors and as potential windows for prevention and promotion.

Third, a critical principle for action in this field concerns the need to work in an inter-sectoral and inter-disciplinary manner. The social determinants of mental health are often inter-related and exist in domains that are traditionally catered for by different sectors (e.g. poverty in the social welfare sector; early childhood education in the education sector; violence prevention in the protection sector) as well as different academic disciplines. For effective action, it is important that actors in different sectors and disciplines work together. Many of the social determinants for mental health also affect physical health, creating opportunities for coalitions providing integrated prevention strategies for both simultaneously.

Fourth, a strength-based approach is vital. For prevention and promotion interventions to be successful and sustainable, it is essential that they build on needs that are felt by local actors as critical to address. Furthermore, prevention and promotion are more compelling and sustainable when they can build on resources that are locally available, or when they build on resources that are valued and can be feasibly introduced in new intervention settings. Petersen *et al*. (2011) in this regard refer to a competency-enhancement approach, and emphasize the importance of recruiting communities as partners in the design, implementation, and evaluation of prevention and promotion interventions. Or, as Allen *et al*. (2014) put it, prevention and promotion ‘needs national policies, but local actions’.

Together, these principles indicate the need for a collaborative and context-sensitive approach (rather than a one-size-fits-all approach), in which (a) detailed assessments of context (to identify key risk, protective, and promotive factors) are coupled with (b) a life-span developmental perspective on how action across the life span can promote mental health and prevent disorders (Tol *et al*. 2013*b*); and (c) in which local communities are empowered to build on their strengths in a participatory manner.

Below examples of evidence-based promotion and prevention interventions in accordance with the different types of intervention are provided: mental health promotion, universal, selective, and indicated prevention. The examples chosen differ in terms of age groups in which they are implemented, and have been implemented in different types of settings in LMICs. These examples were selected to illustrate the breadth of promotion and types of prevention activities across the life span, not necessarily because they have the strongest comparative evidence base in LMICs.

## Mental health promotion: positive parenting in the early childhood period

Early childhood is a critical period in life, in which negative environments may solidify in life-long risks to health and human development, or in which foundations for positive health may be laid. The last decades have seen an upsurge in research in the neurobiological, behavioral, and social sciences, which has greatly increased our understanding of the importance of this period. This research has shown that biology and experience shape each other in a continuous interaction, making the nature *v.* nurture debate outdated. The early childhood period presents a period of plasticity in which the developing nervous system architecture is particularly sensitive to outside influences. In addition, research has shown that relationships with caregivers and others are critical in this period, either for the promotion of health and development or by constituting central risks (Shonkoff & Phillips, 2000). For example, the stress response system is highly malleable in the fetal and early childhood period. Frequent activation of the stress response system, in the absence of supportive caregivers, may result in sustained elevated levels of the stress hormone cortisol and setting the response system in a ‘high alert’ mode. Sustained high levels of cortisol can damage hippocampal functioning, the brain region important for learning and memory. Conversely, children experiencing secure relationships have more controlled stress hormone responses (National Scientific Council on the Developing Child, 2005).

Given that a key objective of global health is to target health inequities within and between populations (Koplan *et al*. 2009), it is not surprising that the early childhood period is viewed as an essential window of opportunity (UNICEF, 2006). The early childhood period provides a chance to ameliorate, for example the pressures of poverty and violence in the home, before they become embedded in children's wellbeing and social functioning. In 2007, an estimated 200 million children in LMICs were not reaching their developmental potential (Walker *et al*. 2007). The identified key risks for suboptimal health and development indicate the value of interdisciplinary and multi-sectoral approaches, in that they reach across several domains: (1) malnutrition and growth (stunting, iodine deficiency, iron-deficiency anemia, intrauterine growth restriction, and prenatal maternal nutrition); (2) maternal mental health (maternal depression and stress); (3) social interaction (inadequate cognitive stimulation, institutionalization, and exposure to violence); and (4) infectious disease and environmental health (malaria, HIV infection, and lead exposure). Breastfeeding and maternal education are protective factors (Walker *et al*. 2011).

The identified importance of inadequate cognitive stimulation and early caregiver relations has spurred interest in parenting interventions in the early years. Such interventions are often implemented by locally trained para-professionals, and have consisted of home visits by community workers or primary health care (PHC) workers, as well as combined nutrition and early stimulation activities through home visits by PHC workers, with most interventions addressing mother–child interaction (for example play, responsive feeding, and communication) (Barry *et al*. 2011).

A systematic review of early childhood development interventions in LMICs identified 11 controlled studies and four assessments of scaled-up programs focused on parenting specifically (Engle *et al*. 2011). In the 11 controlled studies, substantial positive effects were found for child development, including on cognitive or social-emotional development (nine studies), and parent knowledge, home stimulation, and learning activities with children (two studies). Larger effects have been found for interventions focused on both parents and children and for programs with younger and poorer children, Effects for purely information-based and parent-only interventions have been smaller. According to this review, the most effective programs are those that have systematic training methods, a structured and evidence-based curriculum, and opportunities for parental practice while feedback is provided (Engle *et al*. 2011).

## Universal prevention: addressing intimate partner violence

Intimate partner violence is a worldwide highly pervasive phenomenon with well-documented negative consequences for mental health and thereby forms a critical social determinant for mental health. A recent meta-regression of multiple international studies, involving data from 141 studies in 81 countries, found that 30% of women 15 years and older experienced physical, sexual violence, or a combination in their lifetimes. There is considerable regional variation in rates, from 16% in East Asia to 66% in central Sub-Saharan Africa (Devries *et al*. 2013*b*). Among other health consequences, the link with adverse mental health outcomes is well substantiated (Trevillion *et al*. 2012). For example, a systematic review of intimate partner violence and depression found that 9–28% of depressive symptoms can be attributed to intimate partner violence (Beydoun *et al*. 2012). Longitudinal studies have revealed statistically significant relations between intimate partner violence depression and suicide, with an odds ratio of 1.97 for depression across six studies (Devries *et al*. 2013*a*). In the perinatal period specifically there are also consistent relations between intimate partner violence and a range of worse mental health outcomes, including depression, posttraumatic stress disorder (PTSD), and anxiety (Howard *et al*. 2013).

Evidence for the effectiveness of interventions aimed at preventing or reducing intimate partner violence in LMICs has been growing rapidly in the last years – with more recent research focusing on prevention rather than response (Ellsberg *et al*. 2015). From a mental health perspective, the different approaches to prevent or reduce violence can be categorized as universal prevention or selective prevention. For example, a community-based intervention aimed at preventing or reducing intimate partner violence with people regardless of current violence exposure, addresses an important risk factor for mental health at the population level (universal prevention). Interventions in the health care sector with women who have experienced (or who are experiencing) intimate partner violence can be viewed as selective prevention, given that they work with a group at particular risk for the development of mental health problems.

The evidence for prevention of intimate partner violence was summarized – together with evidence on interventions for violence against women more broadly – in a recent special issue in the Lancet. In LMICs, several types of interventions have shown to effectively reduce levels of intimate partner violence, including community mobilization; empowerment training for women and girls; group-based training for women and men; as well as economic empowerment interventions combined with gender equality training (Ellsberg *et al*. 2015).

Of the nine studies included in this review, only one directly examined mental health outcomes. This randomized controlled trial in western Kenya found that unconditional cash transfers were associated both with improvements in female empowerment as well as improvements in several wellbeing indicators (happiness and life satisfaction) and a reduction in perceived stress, depressive symptoms, and in levels of the stress hormone cortisol (Haushofer & Shapiro, 2013). There remains a need for further studies that directly examine how addressing intimate partner violence translates to prevention of mental disorders. Three strands of knowledge support this as a viable research strategy, including (a) aforementioned epidemiological research that shows strong links between intimate partner violence and negative mental health outcomes; (b) evaluations of intimate partner violence response interventions that show simultaneous reductions in intimate partner violence and mental health symptoms (Hegarty *et al*. 2013; Saftlas *et al*. 2014); and (c) rigorous evaluations of intimate partner violence prevention interventions showing concomitant results on reduced intimate partner violence and other health outcomes (e.g. HIV incidence) (Jewkes *et al*. 2008; Wagman *et al*. 2012).

## Selective prevention: school-based interventions for conflict-affected children

Just over one billion children globally live in countries and territories affected by armed conflicts and wars (Machel, 2009). As noted above, exposure to violence constitutes a well-documented risk factor for child development and mental health in LMICs (Walker *et al*. 2011). Exposure to violence has been linked to a range of mental health consequences; the best studied constituting symptoms of PTSD, major depressive disorder, and anxiety disorders (Attanayake *et al*. 2009). However, it is recognized that many children do not show long-term negative mental health impacts of armed conflict, a phenomenon studied under the term resilience. Research on mental health and resilience in children growing up in conflict zones can be particularly informative for the development of selective preventive interventions.

A systematic review of the resilience literature has shown that resilience should not be equated to a simple balance of risk and protective factors with universal relevance. Rather, resilience appears to be determined in complex time- and context-dependent processes. For example, longitudinal studies have shown that the same coping methods had different benefits for mental health depending on whether they were applied in an active phase of conflict or in the post-conflict phase. Similarly, political affiliation appeared protective among conflict-affected youth in Nepal, but the reverse was observed in Bosnian adolescents. Different impacts of protective factors have also been observed depending on gender and developmental stage. Such findings underscore the importance of context-sensitive preventive interventions, rather than the application of similar prevention interventions across diverse socio-cultural contexts (Tol *et al*. 2013*b*). This can be further illustrated by the findings of evaluations of a school-based intervention for conflict-affected children in Burundi, Indonesia, Nepal, and Sri Lanka.

Several randomized controlled trials evaluated the classroom-based intervention (CBI), a manualized group intervention with children aged 8–12 years in 15 sessions over 5 weeks. The CBI combines elements of trauma-focused cognitive behavioral techniques (such as drawings related to adverse events, psychoeducation, and discussion of coping) and creative-expressive activities components (drama exercises, movement, and use of music), aimed at reducing distress and strengthening coping and social support (Macy *et al*. 2003). The CBI was implemented as part of a multi-tiered package of services in several conflict-affected settings. Besides the CBI, this package also included school-based mental health promotion, universal preventive intervention, and treatment interventions (Jordans *et al*. 2010*b*, 2013). A brief screening tool was developed to assess eligibility, including exposure to violence and a perceived lack of coping and social support (Jordans *et al*. 2008, 2009).

Evaluations through cluster randomized trials have shown diverse mental health benefits of the CBI across settings, and identified a complex set of mediators and moderators of intervention benefits. Intervention benefits were found in Burundi (Tol *et al*. 2014), Indonesia (Tol *et al*. 2008), Nepal (Jordans *et al*. 2010*a*), and Sri Lanka (Tol *et al*. 2012). However, intervention benefits were differentiated by both gender and age (Tol *et al*. 2010). Other important moderators of intervention included pre-intervention levels of exposure to conflict-related events (Burundi and Sri Lanka), family-level variables such as household size and family composition (Burundi), and displacement status (Burundi). Moreover, in ongoing sites of conflict (Burundi and Sri Lanka) better outcomes in the control group were identified for some symptom outcomes. Based on these findings, it is clear that context plays a critical role in determining the outcome of preventive interventions.

Although more research is necessary to disentangle the specific mechanisms responsible for these diverse findings, they are consistent with the above-described findings in the epidemiological literature on resilience and mental health. Similar to this epidemiological literature, these intervention studies highlight the critical importance of careful tailoring of interventions to target population and contexts. Further work, for example, is required to study how gender-sensitive interventions may result in more consistent outcomes, and how targeting narrower age groups may change outcomes. However, such changes in narrower targeting need to be weighed against the cost-benefit of reaching a smaller population. Critically, the inclusion of trauma-focused elements in prevention programs may need to be reconsidered in areas of ongoing instability (i.e. the trials in Burundi and Sri Lanka). Removing the trauma-focused element in preventive efforts and providing separate evidence-based treatments for children with more severe psychological difficulties may be indicated here. This direction is supported by a randomized controlled trial by Ager *et al*. (2011) which evaluated an adapted version of CBI with displaced children in northern Uganda without specific trauma-focused elements and found positive impacts on child wellbeing.

## Indicated prevention: preventing postnatal depressive disorder in pregnant women with heightened levels of depressive symptoms

Depression forms a critical global health priority. It is characterized by lowered mood, reduced energy, and decreased activity. Typical symptoms include reduced capacity to enjoy and be interested in activities, severe tiredness after minimum effort, disturbed sleep and appetite, diminished self-esteem, and feelings of worthlessness (World Health Organization, 1990). Of the 7.4% of disease burden caused by mental and substance use disorders globally, depression is responsible for 40.5% – constituting the largest mental health contribution to the global burden of disease (Whiteford *et al*. 2013). Epidemiological research has shown significant variation in prevalence of depression across countries, commonly with an age of onset in young adulthood and an often chronic or relapsing course. Socio-economic disadvantage and exposure to violence are key determinants of depression, and depression itself is associated with increased mortality (e.g. through suicide) (Patel *et al*. 2009). Maternal mental disorders are also associated with worse child health outcomes. A substantial global literature has documented perinatal mental disorders as risks for a broad range of negative child outcomes (including increased risk for premature delivery, lower birth weight, and intrauterine growth restriction; attachment difficulties; impaired physical growth and cognitive development; and child emotional difficulties), some of which can persist into late adolescence (Stein *et al*. 2014).

van Zoonen *et al*. (2014) conducted a systematic review and meta-analysis of interventions to prevent depression globally. Their systematic review focused on randomized controlled trials of prevention interventions (universal, selective, and indicated), with study participants not meeting criteria for major depressive disorder at baseline. Thirty-two studies were identified with a total of 6214 participants, most (30 out of 32) focused on indicated and selective prevention, and 25 of the 32 studies were conducted in the USA and Europe. Fifteen of the studied prevention interventions were based on cognitive behavioral approaches, and five were based on interpersonal group therapy. The quality of the studies was evaluated as relatively high. Overall, the combined interventions reduced the incidence of new cases of depression with 21%. There was low statistical heterogeneity of the effectiveness of prevention interventions between studies. Prevention effectiveness did not differ by prevention type, type of intervention, age group, number of sessions, study country, or target group. Interpersonal group therapy-based interventions showed a lower number needed to treat than cognitive behavioral prevention interventions(7 *v.* 71, respectively) (van Zoonen *et al*. 2014).

One of the studies included in the review concerns a randomized controlled trial of an antenatal psycho-educational intervention to prevent postnatal depression in women in Mexico (Lara *et al*. 2010). Women were recruited at three different types of clinics in Mexico City, and were eligible if they had higher scores on a depression symptom checklist and were over 26 weeks pregnant. The intervention (*Salud Mental de Mamás y Bebés*) consisted of eight weekly 2-h group sessions, covering: (a) education on the perinatal period and risk factors for postnatal depression; (b) reduction of depressive symptoms through increasing positive thinking and pleasant activities, improving self-esteem and self-care; and (c) the provision of a supportive group environment. In addition, both the intervention and control condition, both *n* = 68 at last follow-up, received a self-help book on depression designed for women with limited reading abilities. The study found six new cases of depression in the prevention condition compared to 15 new cases in the control condition. The positive prevention benefits were more pronounced for women with higher depression risks at baseline. Although the study suffered from high attrition rates, it is a promising indication of how depression incidence can be lowered in populations with sub-disordered levels of depression in an urban population in an upper middle-income country.

## Conclusion

This paper aimed to provide a brief overview of the possibilities for effective mental health promotion and prevention of mental disorders in LMICs. It is argued that such efforts should take a central position in the field of global mental health, given limitations to what treatment alone can do to lower the burden of ill mental health. In addition, prevention and promotion efforts can contribute to ensure a more inclusive global mental health field that addresses health inequities through targeting the social determinants of mental health such as poverty, social marginalization, and (gender-based) violence. General principles for promotion and prevention in mental health may be summarized as PaCTS, indicating the importance of a socio-ecological perspective (place); an inter-sectoral and interdisciplinary approach (collaboration), a life span developmental perspective (timing), and a participatory and empowerment approach (strengths).

Mental health promotion and prevention of mental disorders encompass a wide-ranging field. The paper aimed to provide examples to illustrate this breadth by providing examples of promotion and different types of prevention in different life phases: supporting positive parenting in the early childhood period (mental health promotion); community-wide prevention and reduction of intimate partner violence as a critical determinant of ill mental health (universal prevention); school-based preventive interventions for children exposed to conflict-related violence (selective prevention); and preventing the onset of full-scale postnatal depressive disorder in pregnant women with higher levels of depressive symptoms (indicated prevention).

A detailed summary of the evidence supporting all promotion and prevention interventions in mental health in LMICs is beyond the scope of this paper, but interested readers may consult a recent report by Barry *et al*. (2014). Their summary of evidence for prevention and promotion interventions is briefly summarized in Box 2.

Box 2. See *Barry* et al. (2014)

Sufficient evidence in LMICs:

- Home visiting programs for new mothers and their babies integrating mental health promotion within routine pre- and postnatal-care services.
- Screening and prevention for women at risk of postnatal depression.
- Universal social and emotional learning delivered through school-based life-skills programs in primary- and post-primary schools.
- Targeted interventions which enhance resilience, cognitive, and coping skills for children at increased risk of depression and anxiety
- CBIs for vulnerable children (orphaned by HIV/living in areas of conflict/war) improve psychological and social functioning and coping.
- Out-of-school empowerment programs for adolescents/young adults designed to promote youth health through the use of multi-component interventions.
- Screening and brief interventions for problem drinkers by PHC workers.
- Raised tax/price on alcohol; restricted access to alcohol; and bans on alcohol advertising.
- Restricting access to potential lethal means of suicide.

Limited evidence in LMICs:

- Access to pre-schools offering day center and home based educational interventions for children living in poverty – high quality education and parent support focusing on children's emotional, behavioral and social development and parents’ parenting skills and mental wellbeing
- Universal and targeted parenting/family strengthening programs which enhance parenting and family communication skills for promoting children's development
- Community microcredit schemes which incorporate health and education training alongside the provision of credit for income generation

Clearly, there is a need for further strengthening of evidence of preventive interventions, as there is for treatment of a variety of mental disorders in LMICs (Tol *et al*. 2013*a*). It is critical that research capacity and funding in LMICs is strengthened to ensure rigorous prevention research efforts to further this agenda. Nevertheless, the current evidence for promotion and prevention efforts in LMICs supports proactive efforts to enhance wellbeing and prevent suffering before disorder sets in as a critical element of the field of global mental health.
